# Itch induced by peripheral mu opioid receptors is dependent on TRPV1-expressing neurons and alleviated by channel activation

**DOI:** 10.1038/s41598-018-33620-7

**Published:** 2018-10-19

**Authors:** Helvira Melo, Lilian Basso, Mircea Iftinca, Wallace K. MacNaughton, Morley D. Hollenberg, Derek M. McKay, Christophe Altier

**Affiliations:** 1 Inflammation Research Network-Snyder Institute for Chronic Diseases, and Alberta Children’s Hospital Research Institute, Department of Physiology & Pharmacology, Cumming School of Medicine, University of Calgary, Calgary, Alberta Canada; 2 Gastrointestinal Research Group, Department of Physiology & Pharmacology, Cumming School of Medicine, University of Calgary, Calgary, Alberta Canada

## Abstract

Opioids remain the gold standard for the treatment of moderate to severe pain. However, their analgesic properties come with important side effects, including pruritus, which occurs frequently after systemic or neuraxial administration. Although part of the opioid-induced itch is mediated centrally, recent evidence shows that the opioid receptor system in the skin also modulates itch. The goal of our study was to identify the peripherally located transducer mechanisms involved in opioid-induced pruritus. Scratching behaviors in response to an intradermal injection of the mu-opioid receptor (MOR) agonist [D-Ala2, N-MePhe4, Gly-ol]-enkephalin (DAMGO) was quantified in mast cell-, PAR2- and TRPV1-deficient mice or following ablation of TRPV1+ sensory neurons. We found that mast cells−/−, PAR-2−/−, or TRPV1−/− mice still exhibit DAMGO-induced itch responses. However, we show that ablation of TRPV1+ neurons or acute TRPV1 activation by capsaicin abolishes DAMGO-induced itch. Overall, our work shows that peripheral DAMGO-induced itch is dependent on the presence of TRPV1-expressing pruriceptors, but not the TRPV1 channel itself. Activation of these fibers by capsaicin prevents the opioid-induced itch.

## Introduction

Pruritus (or itch) is defined as an unpleasant sensation that induces the reflex to scratch. This sensation is transmitted from the periphery to the brain through the activation of dorsal root and trigeminal ganglion neurons that project to the skin. Itch is a symptom of numerous chronic inflammatory skin or systemic conditions, and is an adverse effect of medications that can often lead to the discontinuation of treatment. Substances that cause itch (pruritogens) are produced endogenously (histamine, kinins, neurotrophins, endothelin-1, bovine adreno medulla (BAM) 8–22, proteases, cytokines or opioids), introduced from the environment (β alanine, Cowhage spicules from the bean plant *Mucuna pruriens*) or delivered as a medication (cloroquine, opioids). These agents produce itch by the activation of different cell types, including keratinocytes, mast cells or a specific subset of DRG neurons^1^. Over the last decade, several receptors and transducers implicated in itch sensation have been identified, including histamine receptors, protease-activated-receptor-2 (PAR-2), members of the Mas-related G-protein coupled receptor family (MrgprD, MrgprC11), the transient receptor potential vanilloid 1 (TRPV1) channel or the transient receptor potential ankyrin 1 (TRPA1) channel^2^.

Furthermore, opioid receptors activated by both endogenous and exogenous opioids are considered as a major class of pruritogen receptors. Itch is a common side effect of opioids used in the treatment of intractable or post-operative pain. Opioids cause itch in >5% of patients, depending on the route of administration: neuraxial injections cause the most symptoms^3^. Indeed, central administration of opioids is the most likely to trigger an itch response, with 30–100% of patients undergoing spinal or epidural administration developing itch.

There is recent evidence that peripheral opioid receptors contribute to itch^4,5^. However, the cell types and opioid receptor subtypes in the periphery that are involved in triggering itch is still a matter of debate^6^. All three opioid receptor subtypes, mu (MOR), delta (DOR) and kappa (KOR), are known to mediate analgesia both centrally and peripherally. Nonetheless, itch appears to be triggered only by mu opioid receptor agonists, delta agonists having no effect and kappa agonists being able to attenuate itch^7^. Accordingly, activation of MOR by intradermal injection of the highly selective MOR agonist DAMGO has been shown to produce an itch response that was inhibited by naloxone-methiodide, a peripherally restricted opioid receptor antagonist^5^.

The MOR has been proposed to be functional in different skin-localized cell types: keratinocytes, fibroblast, immune cells and sensory neurons, which acting together could directly or indirectly, via secreted factors, mediate this itch response. While MOR is particularly enriched in peptidergic TRPV1+ fibers^8,9^ and in spite of the contribution of TRPV1+ fibers in various human itch models using histamine, serotonin or mucunain (cowhage)^10–14^, it remains unclear whether opioid-induced itch is dependent on MOR-expressing dermo-epidermal TRPV1+ sensory neurons.

This study was designed to elucidate the transduction mechanism(s) of opioidergic itch at the periphery using a DAMGO-induced itch model. We employed: (1) mice in which cells (mast cells) or receptors (PAR2, TRPV1) known to be implicated in peripheral itch transduction were absent; and, (2) agents known to acutely stimulate sensory TRPV1+ neurons directly (capsaicin) or to deplete TRPV1+ neurons (resiniferatoxin (Rtx)). We found that ablation of TRPV1-expressing neurons but not mast cell deficiency abrogated peripheral opioid itch. We then report that DAMGO causes an itch response despite deletion of PAR2 or TRPV1. Finally, we show that acute activation of nociceptive fibers by capsaicin, but not AITC, was able to prevent opioid-induced itch. Altogether our results exclude mast cells and PAR2 mediators, but have implications for the understanding and the treatment of opioid-induced itch.

## Materials and Methods

### Animals

C57BL/6, 2–4 month old male mice (25–30 g) were used in the study. Preliminary data showed that both male and female exhibited DAMGO-induced itch similarly, and both sexes were therefore grouped in the analysis. Animals were obtained from Jackson Laboratories and maintained at a controlled temperature (21 ± 1 °C) with free access to food and water and with a 12/12 dark-light cycle. The TRPV1 KO mice were bred at the University of Calgary. The PAR-2 and mast cell (Kit-Wsh/Wsh) knock out and their control littermates were bred at the University of Calgary (Drs. Morley Hollenberg, Wallace MacNaughton and Dr. Derek McKay, respectively). The animal protocol used for this study was approved by the University of Calgary Animal Care Committee and is in accordance with the guidelines of the Canadian Council on Animal Care (AC17-0065).

### Drugs

All drugs used in the study were from Sigma Aldrich, with the exception of naloxone methiodide that was purchased from Santa Cruz Biotechnology. Stock solutions were prepared in dimethyl sulfoxide (DMSO) for capsaicin and resiniferatoxin (RTX), in ethanol for allyl isothiocyanate (AITC), distilled water for [D-Ala^2^, NMe-Phe^4^, Gly-ol^5^]-enkephalin (DAMGO) and PBS for naloxone methiodide and β-alanine. The drugs were used at the following concentrations and routes of administration: DAMGO (200 μM; i.d.), β-alanine 50 mM (i.d), naloxone methiodide 1 mg/kg (s.c.), RTX 0.03, 0.07 and 0.1 mg/kg (s.c.), capsaicin 0.05% (i.d.) and AITC 0.075% (i.d.). Drugs were injected using a volume of 50 µl of solution in the nape of the neck or 20 µl in the cheek. RTX was injected with a volume of 100 μl, as previously described^15^.

### Itch Assay

Male mice were shaved in the back part of the neck 24 h before injection of pruritogens. On the day of the experiments, animals were temporarily restrained and intradermally injected into the nape of the neck. After the intradermal injections, mice were placed in a clear plexiglass container, and videotaped for 30 minutes. The experimenter left the room immediately after the video recorder was started. The number of bouts of scratching was scored by an experimenter blind of the previous treatment received by the mice. The number of scratches during a bout can vary, being one or several. One bout of scratch was defined as mouse lifting the hind paw to the back of neck, until the hind paw was returned to the mouth or to the floor^15^.

To evaluate the algogenic potency of TRPV1 and TRPA1 activation, capsaicin and AITC were injected in the cheek of the mice, and nociceptive wiping behaviors were counted over a 20 minute period.

For the ablation of TRPV1^+^ fibers, animals were pretreated with RTX, an ultrapotent TRPV1 agonist. Mice were treated for three consecutive days with RTX at increasing doses of 30, 70 and 100 µg/kg (s.c.)^16^. One week after the injections, animals were treated with DAMGO or β alanine and the scratching bouts were counted for 30 minutes. Control mice received DMSO diluted in PBS.

Other groups of animals received intradermal injections of capsaicin or AITC and the number of scratching bouts counted during 30 minutes. Animals that received previous injections of capsaicin or AITC then received intradermal injection of DAMGO and the number of scratching bouts recorded for an additional 30 minutes. Control mice received injections of PBS 30 minutes before intradermal injections of DAMGO.

### Hot Plate Test

The hot plate test (BIOSEB) was used to measure the latency of withdraw as described previously^17^. The temperature used was 55 ± 0.5 °C and the basal response time of all animals was measured before experiments (mice that presented baseline latencies of more than 15 seconds were removed from the study). The animals were treated with subcutaneous injections of vehicle (5% of DMSO in PBS) or RTX for three consecutive days and one week later, animals were placed on the heated surface of the plate and the latency of pain response (jumping or licking of the hind paws) was assessed^18^. To avoid damage of the skin tissue, a cut-off time of 30 sec was used.

### Quantification of β-tubulin III (Tuj1) positive fibers

Animals were deeply anesthetized with intraperitoneal injections of Pentobarbital (240 mg/kg) and transcardially perfused with 10 mL of phosphate-buffered saline (PBS) followed by 10 mL of 4% paraformaldehyde (PFA). A skin biopsy of the back part of the neck was collected and fixed in 4% PFA overnight (4 °C), then immerged in 30% sucrose for 24 h (4 °C). The skin was then embedded in OCT (Thermo-Fisher Scientific) and 30 μm slices were cut onto Superfrost slides (VWR International, Mississauga, Ontario). The slides were washed in PBS twice then blocked for 1 hour at room temperature with a PBS solution containing 3% FBS and 0.3% Triton-X. Slides were then immunostained with an anti-β-Tubulin III (Tuj1) antibody (rabbit, 1:500, Sigma) overnight at 4 °C. Following PBS wash, slides were incubated with the Alexa-555-conjugated anti-rabbit (1:1000, Invitrogen) for 1 h at room temperature. Slides were washed in PBS twice and mounted with aqua polymount (Polyscience). Z-stack confocal images of the skin sections were acquired on a Zeiss LSM-510 Meta inverted microscope using a 40X water immersion objective. Images were analysed using the Zeiss LSM Image Browser software. Z stacks were processed as maximum intensity projection images and the numbers of Tuj1+ fibers were counted across randomly selected sections. The number of fibers from each section was summed up and the density was obtained by dividing the number of fibers by the length of the epidermal surface (μm). Density is expressed as number of fibers per 100 μm of epidermal surface.

### Statistical Analysis

Data were analyzed using 5.0 Graphpad Prism software. Results are shown as Whiskers box min-mean-max and an individual square depicts each individual mouse. Statistical analysis was performed by using Kruskall-Wallis followed by post-hoc Dunn’s test, or Mann Whitney U test.

## Results

### Activation of peripheral MOR by DAMGO induces itch

**Figure 1:**
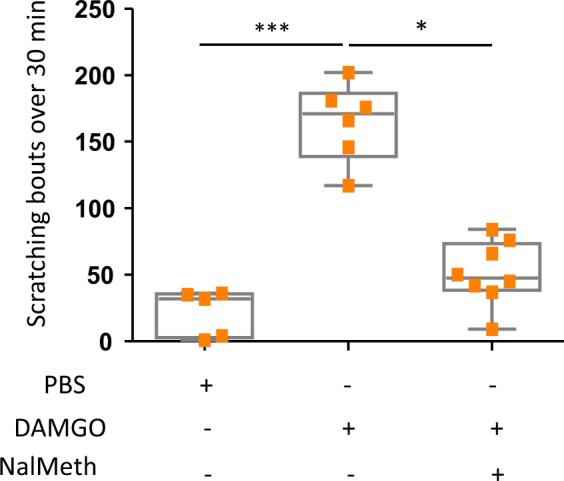
Scratching behavior induced by DAMGO is suppressed by a pre-treatment with naloxone-methiodide. Scratching bouts evoked by intradermal injections of either PBS (n = 5) or DAMGO (200 µM) in the nape of the neck 30 min after a pre-treatment with PBS (n = 6) or naloxone methiodide (1 mg/Kg, s.c, n = 8)). Results are represented as Whisker-dot plot (Min-Mean-Max) and each symbol represents an individual mouse. Statistical analysis were performed using Kruskall-Wallis followed by post-hoc Dunn’s test (***p < 0.01 WT + DAMGO versus WT + PBS and *p < 0.05 represents WT + DAMGO versus NAL + DAMGO).

To assess the effect of activation of peripheral MOR on itch, we injected DAMGO intradermally in the back part of the mouse neck. As shown in Fig. 1, DAMGO triggers a marked itch response compared to PBS, with 150 cumulative scratching bouts within the 30 minutes of DAMGO injection (p < 0.001). This itch response, dependent on the activation of peripheral MOR, was inhibited by treatment with the peripherally restricted opioid receptor antagonist Naloxone-Methiodide (p < 0.001). Our results therefore demonstrate that the intradermal administration of DAMGO induces itch in mice, via a peripheral MOR, naloxone-sensitive mechanism.

### Mast cells do not contribute to DAMGO-induced itch

**Figure 2:**
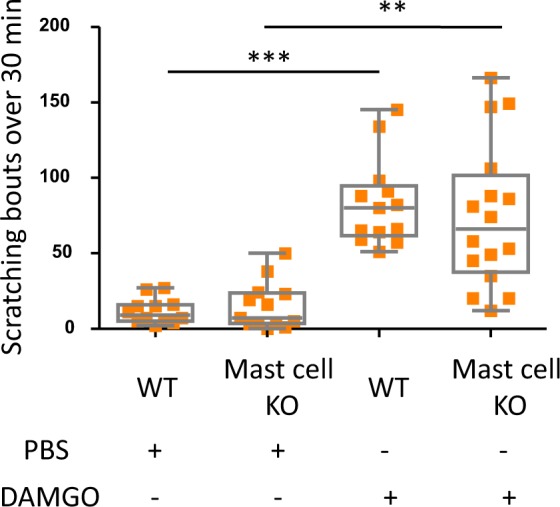
DAMGO induces itch in mast cell-deficient mice. Scratching bouts evoked by intradermal injections of either PBS or DAMGO (200 µM) in WT (PBS; n = 12, DAMGO; n = 13) and mast cell-knockout (KO) mice (PBS; n = 14, DAMGO; n = 16). Results are represented as Whisker-dot plot (Min-Mean-Max) and each symbol represents an individual mouse. Statistical analysis were performed using Kruskall-Wallis followed by post-hoc Dunn’s test (***p < 0.001 WT + DAMGO versus WT + PBS and **p < 0.01 represents mast cell KO + PBS versus mast cell KO + DAMGO).

Mast cells have been shown to participate in the pathophysiology of peripheral itch by secreting histamine in response to various stimuli^1^, including the MOR agonist morphine^19^. To test whether DAMGO activation of MOR-expressing mast cells could be implicated in this effect, we used mast cell deficient mice, Kit-Wsh/Wsh and their control littermates. As shown in Fig. 2, both mast-cell deficient mice and WT littermates exhibit itch in response to locally administered DAMGO, suggesting that peripheral MOR-induced itch is mast cell-independent. Given that mast cell-released histamine is an important mediator of peripheral itch, our results indicate that DAMGO may elicit an itch response via direct activation of peripheral itch-sensing neurons.

### PAR-2 activation is not involved in DAMGO-induced itch

**Figure 3:**
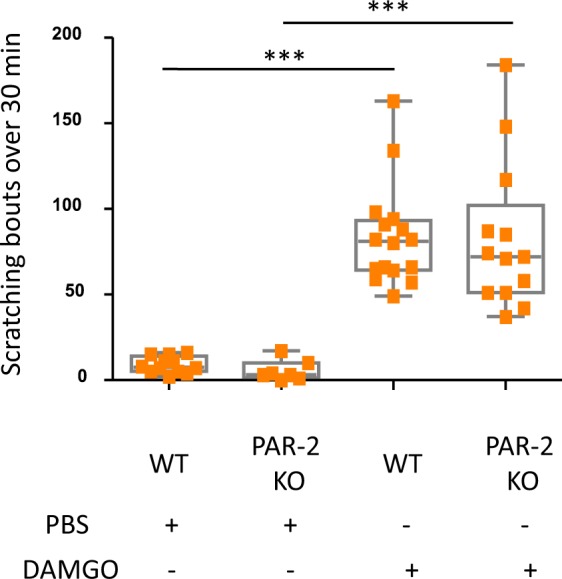
DAMGO induces itch in PAR-2 deficient mice. Scratching bouts evoked by intradermal injections of either PBS or DAMGO (200 µM) in WT (PBS; n = 12, DAMGO; n = 16) and PAR-2-deficient mice (PBS; n = 7, DAMGO; n = 13). Results are represented as Whisker-dot plot (Min-Mean-Max) and each symbol represents an individual mouse. Statistical analysis were performed using Kruskall-Wallis followed by post-hoc Dunn’s test (***p < 0.001 WT + DAMGO versus WT + PBS and ***p < 0.001 represents PAR-2 KO + PBS versus PAR-2 KO + DAMGO).

PAR-2 is a G protein-coupled receptor, which is activated by protease-mediated receptor cleavage. Its activation on peripheral nerve endings has been shown to induce both neurogenic inflammation and itch^20,21^. Furthermore, a functional interaction between PAR-2 and opioid receptors has been proposed by the fact that naltrexone, an opioid receptor antagonist, is able to reverse PAR-2 activation-induced itch in mice^22,23^. To determine if PAR-2 contributes to the itch response triggered by DAMGO, we tested PAR-2 deficient mice in our model. As shown in Fig. 3, DAMGO administration induced equivalent itch behavior in both PAR-2 deficient mice and WT littermates, suggesting a PAR2-independent mechanism by which DAMGO elicits itch at peripheral nerve endings.

### TRPV1 is not involved in DAMGO-induced itch

**Figure 4:**
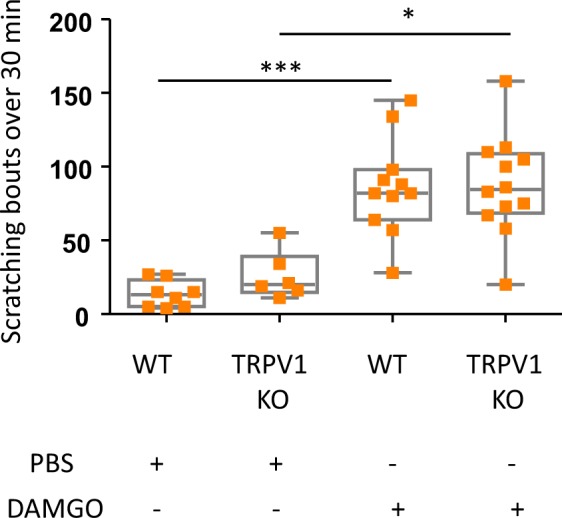
DAMGO induces itch in TRPV1 deficient mice. Scratching bouts evoked by intradermal injections of either PBS or DAMGO (200 µM) in WT (PBS; n = 8, DAMGO; n = 11) and TRPV1 deficient mice (PBS; n = 6, DAMGO; n = 12). Results are represented as Whisker-dot plot (Min-Mean-Max) and each symbol represents an individual mouse. Statistical analysis were performed using Kruskall-Wallis followed by post-hoc Dunn’s test (***p < 0.001 WT + DAMGO versus WT + PBS and *p < 0.05 TRPV1 KO + PBS versus TRPV1 KO + DAMGO).

One of the main nociceptive transducers that plays a role in peripheral itch is the TRPV1 channel. TRPV1 has been particularly involved in histamine induced-itch^24^, and in chronic kidney disease-induced pruritus (CKD-iP)^25^. Interestingly, opioid signaling also contributes to CKD-iP, as naltrexone was found to provide itch relief in both pre-clinical^25,26^ and clinical studies^27^. We thus asked whether TRPV1, downstream of MOR activation by DAMGO, could participate in eliciting peripheral opioid-induced itch. As shown in Fig. 4, in TRPV1-deficient mice, locally administered DAMGO induced a scratching response that was equivalent to WT littermates. Thus, like the mast cells and PAR2, TRPV1 does not participate in the transduction of itch induced by DAMGO.

### Peripheral DAMGO-induced itch is elicited by TRPV1-expressing primary afferents

**Figure 5:**
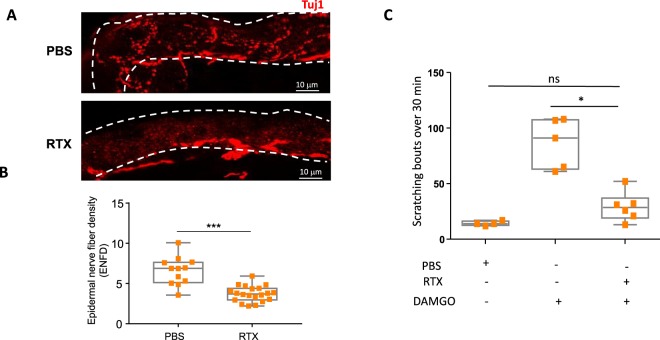
TRPV1-expressing C-fibers are required for DAMGO-induced itch (**A**) Representative images and (**B**) quantification of intraepidermal Tuj1+ nerve fiber density per 100 μm of skin tissue, between PBS and RTX (three consecutive injections, one day apart: 30, 70 and 100 µg/mL) treated mice. Dotted line delineates the epidermis. Statistical analysis were performed using Mann Whitney U test (***p < 0.001 PBS versus RTX). (**C**) Scratching bouts evoked by intradermal injections of either PBS or DAMGO (200 µM) in WT (PBS; n = 4, DAMGO; n = 5) and RTX-treated mice (n = 6). Results are represented as Whiskers-dot plot (Min-Mean-Max) and each symbol represents an individual mouse. Statistical analysis were performed using Kruskall-Wallis followed by post-hoc Dunn’s test (*p < 0.05 PBS + DAMGO versus RTX + DAMGO).

Different subsets of sensory neurons mediate peripheral itch, in particular, slow conducting unmyelinated C fibers that can be divided into two groups: (1) non-peptidergic C-fibers, that express Mas-Related G-protein coupled Receptors (Mrgpr), major mediators of peripheral-itch^28^ and are characterized by their high affinity binding to the lectin IB4 and, (2) peptidergic C-fibres, characterized by expression of Substance-P and Calcitonin-Gene Related Peptide along with the TRPV1 channel^29^. To test which subset of sensory neuron is implicated in DAMGO-induced itch, we ablated TRPV1-positive fibers with repeated administration of Resiniferatoxin (RTX), an ultra-potent TRPV1 agonist. This approach resulted in the loss of TRPV1-expressing fibers as attested by a loss of sensitivity to noxious heat (55 °C) using the hot plate test (Suppl. Fig. 1A) and a decrease in the epidermal nerve fiber density (Fig. 5A,B)^30^. Ablation of these RTX sensitive fibers was able to abrogate DAMGO-induced scratching, demonstrating the role of TRPV1-expressing neurons in the opioid-induced itch response. However, itch induced by the β alanine-activated MrgprD receptor, which is expressed in non peptidergic TRPV1 negative fibers of the skin, was maintained following RTX treatment (Suppl. Fig. 1B)

### Activation of TRPV1 by capsaicin attenuates DAMGO-induced itch

**Figure 6:**
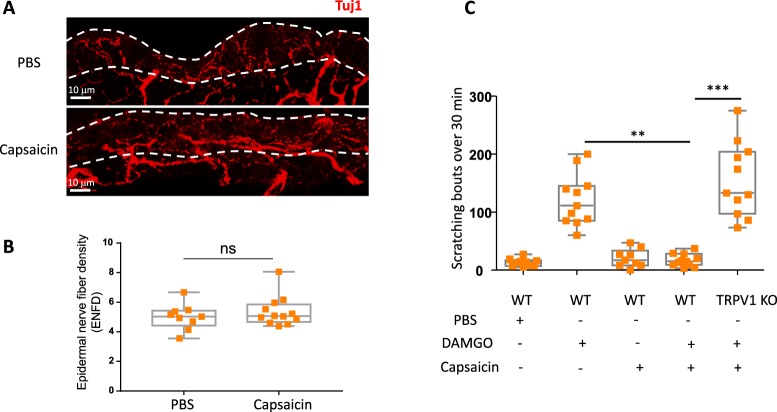
Acute injection of capsaicin prevents DAMGO–induced itch. (**A**) Representative images and (**B**) quantification of intraepidermal Tuj1+ nerve fiber density per 100 μm of skin tissue, between PBS and capsaicin (0.05%) treated mice. Dotted line delineates the epidermis. Statistical analysis were performed using Mann Whitney U test. (**C**) Scratching bouts evoked by intradermal injections of either PBS or DAMGO (200 µM) in WT (PBS; n = 10, DAMGO; n = 11), capsaicin pre-injected mice (PBS; n = 9, DAMGO; WT n = 11 and TRPV1 deficient mice n = 11). Results are represented as Whisker-dot plot (Min-Mean-Max) and each symbol represents an individual mouse. Statistical analysis were performed using Kruskall-Wallis followed by post-hoc Dunn’s test (**p < 0.01 WT + DAMGO versus capsaicin + DAMGO and ***p < 0.001 capsaicin + DAMGO in WT versus TRPV1 KO mice).

It is well established that itch sensation can be reduced by painful stimuli, such as the scratching response on the skin^31^. Moreover, chemical noxious stimuli can also have antipruritic effects, and capsaicin has been demonstrated to attenuate scratching induced by pruritogens like histamine, PAR-2 agonist or substance P^32^. To test the effect of activating nociceptive chemosensors on MOR-induced peripheral itch, we assessed scratching behavior in response to DAMGO after local injections of either capsaicin (0.05%) or AITC (0.075%), substances that cause pain by activating TRPV1 or TRPA1 channels, respectively. Importantly, injection of capsaicin on the rostral back of mice 30 minutes before DAMGO administration did not affect the density of epidermal skin fibers assessed via immunostaining of Tuj1 in the skin of untreated and treated animals (Fig. 6A,B). Nevertheless, as shown in Fig. 6C, pre-treatment with intradermal capsaicin prevented DAMGO-induced itch. This effect was dependent on the specific activation of TRPV1, as capsaicin failed to prevent DAMGO-induced itch in TRPV1 KO mice (Fig. 6B). In contrast, the TRPA1 agonist AITC that elicited nociceptive wiping behaviors similar to capsaicin, following injection into the mouse cheek (Suppl Fig. 2A), did not alter the DAMGO-induced itch (Suppl Fig. 2B). Thus MOR-induced peripheral itch was specifically inhibited via the concurrent activation of TRPV1 channels on sensory neurons.

## Discussion

Our findings provide key information regarding the mechanisms of peripheral opioid induced itch. We report that whilst neither mast cells nor the pruritogen receptor, PAR2, play a role in itch caused by the local activation of the MOR by intradermal DAMGO, nerve fibers emanating from TRPV1-expressing neurons, and eliminated by chronic resiniferatoxin treatment, are essential for the itch response. Further we found that the co-stimulation of the MOR-responsive nerves with the TRPV1 agonist, capsaicin (but not by the TRPA1-activating agonist, AITC), was able to eliminate the DAMGO-triggered itch response.

What singles out our findings from the data of others is that, although eliminating the TPRV1 sensory fibers abolishes MOR-induced itch, the presence of the fibers devoid of TRPV1 in the TRPV1-null mice still enables MOR signalling and does not affect itch response. It is well established that TRPV1 is expressed in a large subset of itch-sensing neurons that are activated by a variety of pruritogens^24^. However, TRPV1 does not transduce itch for all pruritogens that activate TRPV1+ pruriceptive neurons^13^. For instance, histamine induces itch through TRPV1 activation, while serotonin or endothelin-1 do not, even though their respective receptors are expressed on TRPV1+ neurons. Therefore, our results show that, unlike the histamine receptor, that drives itch through TRPV1 signaling, but like serotonin and endothelin, MOR must engage different ion channels on TRPV1+ pruriceptive neurons. Nevertheless, this fiber-dependent, TRPV1-independent DAMGO/MOR-induced itch signal is blocked by the concurrent activation of the TRPV1 channel by capsaicin, that otherwise leaves the sensory fibers intact. Thus algogenic function of TRPV1 on nociceptors can reciprocally attenuate MOR-induced itch. While it is likely that activation of calcium entry via the TRPV1 channel may cause a counter-regulatory effect on MOR signaling, the mechanism for this inhibitory action of TRPV1 channel remains to be determined. Recently, Scherer *et al*. reported a regulatory role of TRPV1 on MOR function^33^, by blocking receptor phosphorylation without affecting G protein coupling. It will thus be of interest to test whether such molecular TRPV1-MOR crosstalk contributes to opioidergic itch modulation.

We are confident that our RTX approach used to deplete TRPV1-expressing cells is selective for neurons, as a recent study reported the lack of TRPV1 expression in immune cells, including macrophages, T cells or dendritic cells that could modulate itch^34^. In agreement with our results, TRPV1-expressing fibers represent the main subset of sensory neurons encoding peripheral itch^2^. Deletion of these fibers by chronic neurotoxic doses of capsaicin^24^, or by the neuron-specific expression of cytotoxic diphteria toxin expressed under control of the TRPV1 promoter^35^, completely abrogated itch in response to multiple pruritogens, including histamine, endothelin-1, chloroquine, or PAR-2 agonists in mice. Importantly, these observations have been confirmed in humans, as ablation of TRPV1 fibers with capsaicin robustly inhibits histaminergic and non-histaminergic itch in healthy volunteers^36,37^. Our findings indicate that TRPV1-expressing neurons are pivotal sensory cells that mediate the itch response to peripheral MOR agonists. Activation of these neurons by capsaicin negatively regulates MOR-induced itch.

While we show that DAMGO-induced peripheral itch is not dependent on the presence of mast cells or activation of PAR2, our study does not rule out keratinocytes as another player. Indeed, keratinocytes have been shown to express opioid receptors in both skin from control and patients suffering from atopic dermatitis^4^, and thus were implicated in peripheral itch^1,38^. That said, keratinocytes do not respond to vanilloids^39^ and thus would not be thought of as a target for the counter-regulatory itch-inhibiting action of capsaicin.

We can’t ascertain that the concentration of DAMGO that was used in our study reflects the concentration of opioid reaching the skin following exogenous opioid administration or during immune-derived opioid release. However, this concentration was shown to be the lowest one able to trigger peripheral itch in mice^5^, and thus validate this model of peripheral itch induced by MOR activation. Indeed, DAMGO is a highly specific MOR agonist, with little affinity for the DOR or the KOR^40^. Our data are in line with previous ones reporting the role of MOR in peripheral itch^5^ and comfort the findings that MOR and TRPV1 co-express in a specific subset of DRG neurons^8^. Further work will be needed to address how the MOR system can activate TRPV1+ pruriceptors given that a coupling of MOR to Gi/o protein has been well established at the soma of DRG neurons or at their presynaptic nerve terminals^41^.

In peripheral itch, opioid agonists such as morphine or methadone (but not fentanyl or oxymorphone) cause local itch, and some opioids, like morphine or codeine, were suggested to promote release of histamine from mast cells in a non immunological manner^42,43^. Our results using Kit-Wsh/Wsh mast cell deficient mice, show that DAMGO-induced itch is independent of the presence of mast cells. These results are in line with a recent paper showing that morphine, but not DAMGO, activates mast cell to release histamine, through the activation of the atypical opioid receptor, MRGPRX2^19^.

We also show that DAMGO-induced itch is independent of the presence of PAR-2^21,44^, a receptor whose activation by the synthetic agonist SLIGRL^45^, or endogenous proteases^20,46,47^, contribute to pathological itch. Interestingly however, PAR-2-dependent itch was shown to be inhibited by the opioid receptor antagonist naloxone, in response to the PAR2 activating peptide and in a model of local dermatophyte-induced itch^23,48^, indicating a functional relationship between PAR-2 activation and opioids in itch conditions. Further work may determine whether opioid receptor activation could be a downstream target of PAR-2 induced-itch.

Opioid receptor antagonists have long be known to treat numerous pruritic conditions^49^, including uremic or cholestatic pruritus, suggesting a role for endogenous opioids in triggering itch^50,51^. A recent study highlighted β-2 microglobulin as an important molecule that could mediate uremia-induced itch^26^. β-2 MG is a major histocompatibility complex (MHC)-I associated molecule, which is increased in the blood and skin of uremic patients. Interestingly, the authors showed that intradermal injection of β-2 MG induced itch in mice in a naloxone-dependent process. Similar to our study, the β-2 MG-induced itch was histamine and PAR2-independent, and desensitization of TRPV1-expressing fibers attenuated the response. Therefore, opioid signaling seems to be at the crossroad of multiple peripheral itch pathways.

It can be noted that capsaicin has been used to treat pruritic conditions^52,53^. Capsaicin is able to act acutely, by evoking pain signaling and thus causing segmental reduction of itch in the spinal cord^13,54^. Furthermore, long term relief of itch in clinical settings is achieved through defunctionalization of TRPV1+ pruriceptive fibers at the periphery^55,56^. Recent findings have also shown that TRPV1 activation could block opioid-dependent phosphorylation of MOR while leaving G protein signaling intact^33^. In the event that phosphorylation of MOR in response to agonists and recruitment of β-arrestin signaling contribute to opioidergic itch, activation of TRPV1 may cause a molecular bias towards G protein signaling, thereby reducing itch response. Overall, our findings could be explained by an alternative non-exclusive mechanism whereby activation of TRPV1 in MOR-expressing neurons alters MOR signaling, very likely via a TRPV1-mediated calcium signaling or an alteration in MOR-effector/β-arrestin coupling.

Overall, our work highlights peripheral MOR on TRPV1-expressing neurons, as important mediators of peripheral itch. This MOR-induced itch, which we show is TRPV1+ neuron-dependent, but independent of mast cells, PAR-2 and TRPV1 channels *per se* is nonetheless subject to negative regulation by TRPV1 signaling. Our data also reinforce the concept that TRPV1+ epidermal fibers represents the target of many pruritogens, including MOR agonists.

## Electronic supplementary material

Supplementary Figures
