# Supplementary material for: Itch induced by peripheral mu opioid receptors is dependent on TRPV1-expressing neurons and alleviated by channel activation
Source: Sci Rep. 2018 Oct 19;8:15551. doi: 10.1038/s41598-018-33620-7 (PMC6195532; doi:10.1038/s41598-018-33620-7)
Supplement: Supplementary file 1 — Supplementary Figures [file 41598_2018_33620_MOESM1_ESM.pdf]

Supplementary Information:

Itch induced by peripheral mu opioid receptors is dependent on TRPV1-expressing neurons and alleviated by channel activation

Helvira Melo, Lilian Basso, Mircea Iftinca, Wallace K. MacNaughton, Morley D. Hollenberg, Derek M. McKay Christophe Altier

Supplementary Figure legends:

Supplementary Fig. 1. Ablation of TRPV1+ fibers by RTX does not prevent β-alanine – induced itch.

(A) Withdrawal latency time (s) to 52°C hot plate test in PBS (n=3) or RTX (n=6) treated animals. (B) Scratching bouts evoked by intradermal injections of β-alanine (50mM) in the nape of the neck of naive (n=8) or RTX treated mice (n=5) compared to PBS control (n=6). Statistical analysis was performed using Kruskall-Wallis followed by post-hoc Dunn's test.

Supplementary Fig 2. Pre-treatment with AITC does not alter itch induced by intradermal injection of DAMGO in male and female mice.

(A)Nociceptive wiping behaviors counted over 20 minutes after intradermal injection of capsaicin (0.05%) or AITC (0.075%) in the mouse cheek (n=6). Statistical analysis were performed using Mann Whitney U test. (B) Scratching bouts counted over 30 minutes after intradermal injections of either PBS or DAMGO (200μM) in mice pre-treated with (PBS; n=10) or AITC (0.075%, n=8). Statistical analysis was performed using Kruskall-Wallis followed by post-hoc Dunn's test (***$p<0.001$; PBS versus AITC+DAMGO and **$p<0.05$; AITC+PBS versus AITC+DAMGO).

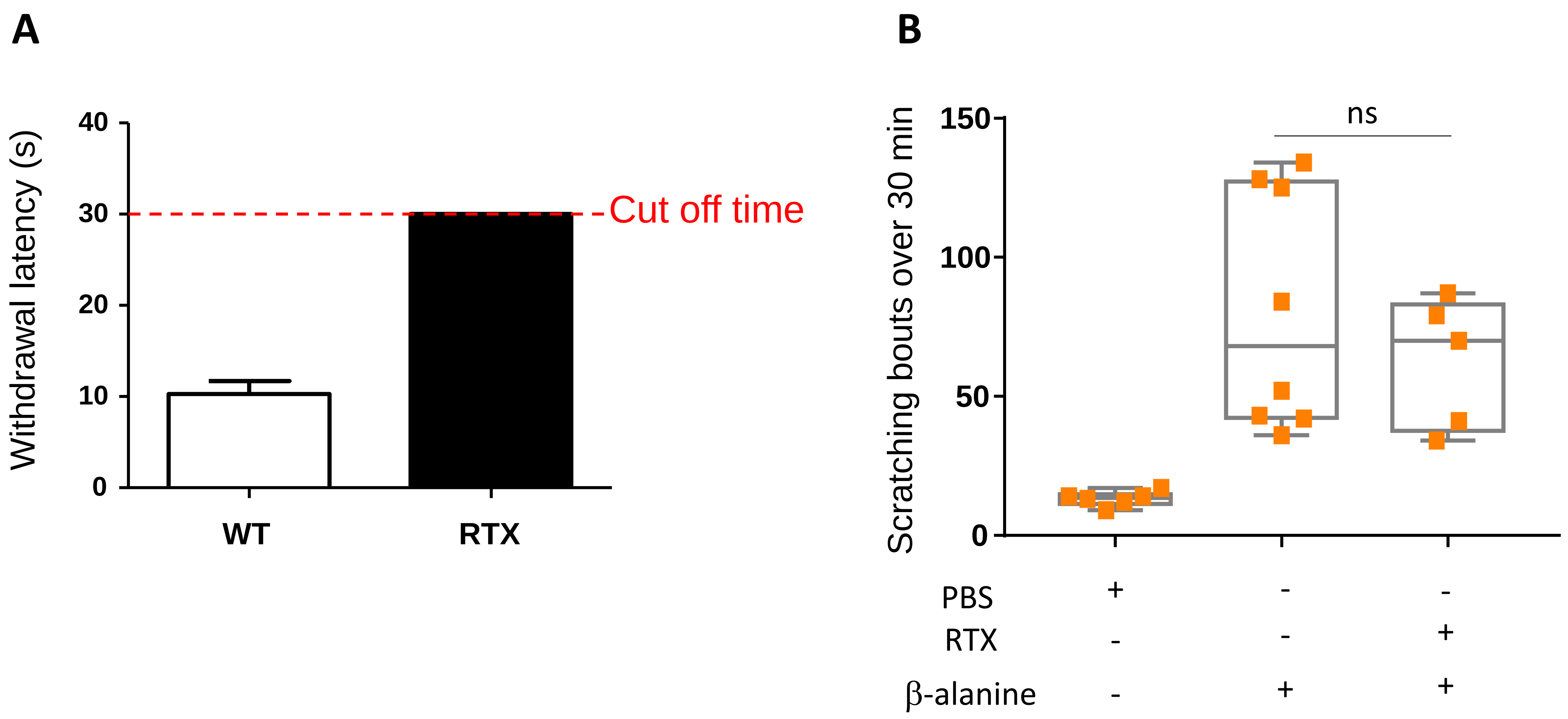

Supplementary Fig. 1

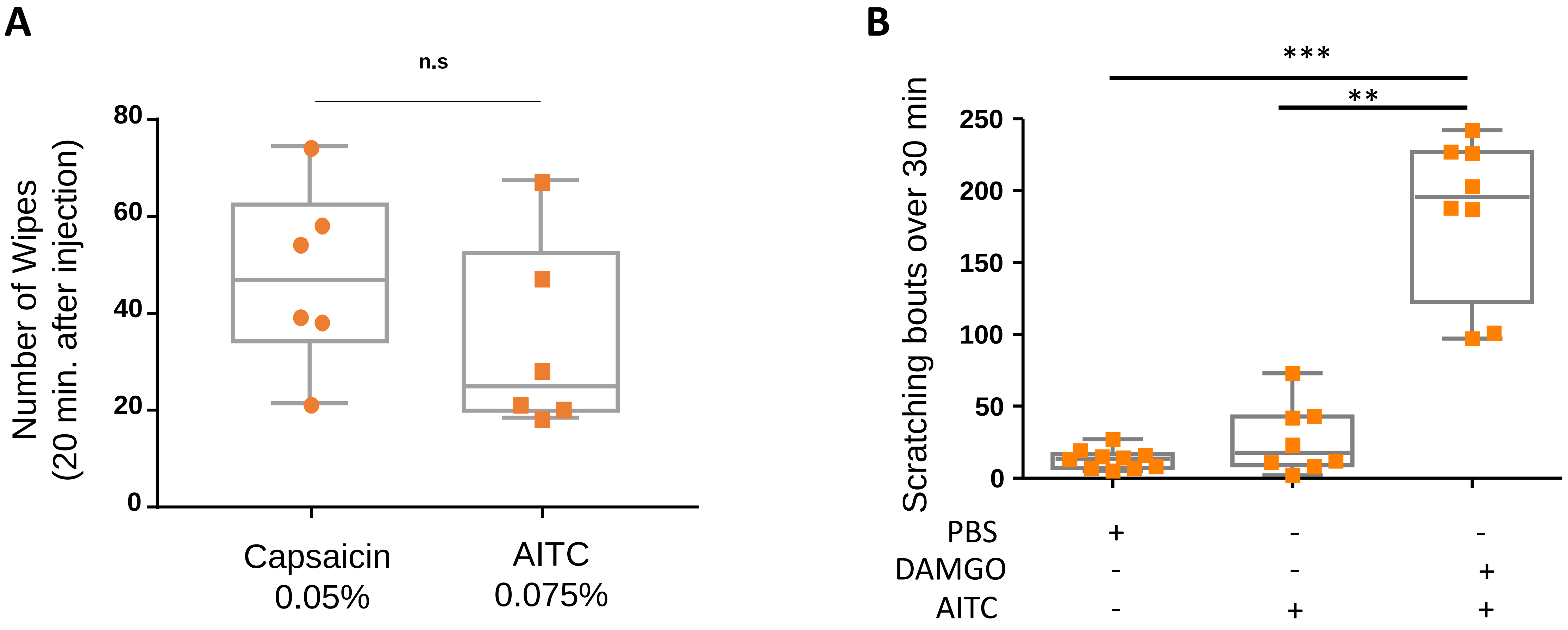

Supplementary Fig. 2
